# Economy of Effort or Maximum Rate of Information? Exploring Basic Principles of Articulatory Dynamics

**DOI:** 10.3389/fpsyg.2019.02469

**Published:** 2019-11-22

**Authors:** Yi Xu, Santitham Prom-on

**Affiliations:** ^1^Department of Speech, Hearing and Phonetic Sciences, University College London, London, United Kingdom; ^2^Department of Computer Engineering, King Mongkut’s University of Technology Thonburi, Bangkok, Thailand

**Keywords:** maximum rate of information, economy of effort, stiffness, peak velocity, target approximation

## Abstract

Economy of effort, a popular notion in contemporary speech research, predicts that dynamic extremes such as the maximum speed of articulatory movement are avoided as much as possible and that approaching the dynamic extremes is necessary only when there is a need to enhance linguistic contrast, as in the case of stress or clear speech. Empirical data, however, do not always support these predictions. In the present study, we considered an alternative principle: *maximum rate of information*, which assumes that speech dynamics are ultimately driven by the pressure to transmit information as quickly and accurately as possible. For empirical data, we asked speakers of American English to produce repetitive syllable sequences such as wawawawawa as fast as possible by imitating recordings of the same sequences that had been artificially accelerated and to produce meaningful sentences containing the same syllables at normal and fast speaking rates. Analysis of formant trajectories shows that dynamic extremes in meaningful speech sometimes even exceeded those in the nonsense syllable sequences but that this happened more often in unstressed syllables than in stressed syllables. We then used a target approximation model based on a mass-spring system of varying orders to simulate the formant kinematics. The results show that the kind of formant kinematics found in the present study and in previous studies can only be generated by a dynamical system operating with maximal muscular force under strong time pressure and that the dynamics of this operation may hold the solution to the long-standing enigma of greater stiffness in unstressed than in stressed syllables. We conclude, therefore, that maximum rate of information can coherently explain both current and previous empirical data and could therefore be a fundamental principle of motor control in speech production.

## Introduction

### Hypo- and Hyper-Articulation and Physiological Effort

To produce a speech sound, the vocal tract needs to be shaped in such a way that appropriate acoustic patterns are generated to allow listeners to identify the intended phonetic category. The shaping of the vocal tract takes time, and the quality of the sound produced may therefore depend on how much time is available for each sound. If there is too little time, the articulators may not be able to move in place, resulting in undershooting the target. This is known as the undershoot model (Lindblom, 1963), and it was based on the finding that vowel formants in a symmetrical /d_d/ consonant context vary with the duration of the vowel. Lindblom (1963) attributes such reduction to a constraint on the speed of articulatory movement. He further shows that duration is the main determinant of the reduction, whether the duration change is due to speech rate or degree of stress, i.e., stress affects vowel reduction only indirectly, i.e., through duration. This undershoot model, however, was questioned by a number of subsequent studies. Also examining formant movements of vowels surrounded by consonants, Gay (1978: 228) concludes that: “differences in vowel duration due to changes in speaking rate do not seem to have a substantial effect on the attainment of acoustic vowel targets.” Based on acoustic and electromyographic data, Harris (1978: 355) concludes that the effects of changing stress and speaking rate are independent of each other and that this is in support of the “extra energy” model: “extra energy is applied to the stressed vowel, with the result that it lasts longer, and the signals to the articulators are a little larger, so that the vowel is further from a neutral vocal tract position.” An important methodological feature shared by both Gay (1968) and Harris (1978), however, is that the target vowels examined are surrounded by /p/, a consonant known to conflict little with the vowel articulation as far as the tongue is concerned. As pointed out by Moon and Lindblom (1994: 41), “according to the undershoot model, no formant displacements would be expected for adjacent vowels and consonants with identical, or closely similar, formant values.”

To address the conflicting data reported subsequent to Lindblom (1963) as mentioned above, Moon and Lindblom (1994) examined the formant frequencies of front vowels in English in a /w_l/ frame at varying durations in clear and casual speaking styles. The large articulatory distances between the vowel and the surrounding consonants indeed led to greater duration dependencies in the formant values than in previous studies, thus reaffirming the early finding of Lindblom (1963). On the other hand, however, they also found that the duration dependency of undershoot is reduced in clear versus normal citation speech, which has led to their further conclusion that undershoot is a function of not only vowel duration and locus–target distance but also the rate of formant change, which is presumably faster in clear than in normal speech. The finding that velocity of articulatory movement is greater in clear speech has led to the theorization, known as the H&H theory, that “within limits speakers appear to have a choice whether to undershoot or not to undershoot,” and that “avoiding undershoot at short segment durations entails a higher biomechanical cost” (Lindblom, 1990: 417). For this reason, energy saving is proposed as a core mechanism of undershoot in addition to time pressure.

Note, however, that energy saving and time pressure are two very different needs, each with very different implications for the explanation of undershoot. Undershoot due to energy saving would entail an effort reduction that slows down the articulatory movements. Undershoot due to time pressure, in contrast, would entail articulatory movements that are as fast as possible (driven by maximum articulatory force) before being cut short by premature termination. Of the two scenarios, the latter is much less explored than the former, not only because of the popularity of the H&H theory but probably also because the implication of the maximum speed of articulation is too extraordinary to be even worth contemplating. As asserted in Lindblom (1983: 219), “in normal speech the production system is rarely driven to its limits.”

### Maximum Speed of Articulation: Is It Really Never Approached?

But there is already evidence that speech production is often driven to its extremes as far as speed of articulation is concerned. Based on a comparison between normal speech and Maximum Repetition Rate (measured in phones per second), Tiffany (1980: 907) concludes that “in some senses we normally speak about ‘as fast as we possibly can,’ at least in the production of full canonical utterances.” Janse (2004) has compared the perceptual word processing speed of Dutch sentences sped up in two ways: (1) by asking the speaker to speak faster, and (2) by computationally time-compressing sentences originally produced at a normal rate. She finds that the perceptual reaction time to the natural-fast sentences is slower than to the computationally time-compressed normal utterances. This finding is further confirmed by Adank and Janse (2009), who show that perception of natural-fast speech has much lower recognition accuracy than does that of time-compressed speech. One likely explanation is that synthetically sped-up speech is still well within the processing speed of the human perceptual system, while naturally speeding up speech forces speakers to reach too many dynamic limits of articulation, and the resulting undershoot is serious enough to impair the quality of information transmission. If this interpretation is right, it is likely that some dynamic limits of articulation are already approached at normal speaking rate. The evidence seen in these studies is somewhat indirect, however. Attempts to more directly compare the performance space of speech and non-speech articulatory movements by using kinematic measurements have produced inconclusive results (Nelson et al., 1984; Perkell et al., 2002). More direct evidence is seen only in the case of F_0_ production, where it is shown that the maximum speed of pitch change is indeed often approached (Xu and Sun, 2002; Kuo et al., 2007; Xu and Wang, 2009). It is therefore necessary to establish more directly than before whether dynamic limits of segmental production are also frequently reached.

### Hyper-Articulation: Does It Overshoot the Target?

It is unlikely, of course, that dynamic limits of articulation are reached *all the time* in each speech utterance, as it is well known that segment and syllable durations change frequently due to various linguistic functions (Lehiste, 1972; Turk and Shattuck-Hufnagel, 2007; Xu, 2009). In many instances, e.g., at domain-final locations where lengthening regularly occurs (Lehiste, 1972; Klatt, 1975; Nakatani et al., 1981; Edwards et al., 1991; Turk and Shattuck-Hufnagel, 2007), phonetic units show durations that well exceed the amount of time needed for achieving their targets. In those cases, does target overshoot (i.e., going beyond the underlying target) happen? For example, would there be a hyperspace effect for vowels, showing an F1–F2 distribution that exceeds their canonical space? The H&H theory may suggest that this would indeed happen, as an enlarged vowel space would enhance phonological contrast (Lindblom, 1990). A hyperspace effect was reported by Johnson et al. (1993a), although they did not link it to durational changes. Whalen et al. (2004), however, failed to replicate the hyperspace effect.

Also, the notion of overshoot may not be fully compatible with the notion of phonetic target. Lindblom (1963: 1773) defines a vowel target as “an invariant attribute of the vowel” that is “specified by the asymptotic values of the first two formant frequencies of the vowel and is independent of consonantal context and duration.” A similar target concept is also seen in the task dynamic model, which assumes that for each articulatory gesture, there is an equilibrium point to which the articulatory state will relax by the end of the gestural cycle (Saltzman and Kelso, 1987; Saltzman and Munhall, 1989). The equilibrium-point hypothesis (Perrier et al., 1996a, b) also assumes a target that is invariant. In none of these models can the target itself be exceeded. There are also models that assume that phonetic targets are not fixed but have variable ranges that can be described as area targets as opposed to point targets (Keating, 1990; Guenther, 1994); this may potentially allow target overshoot. However, the very reason for proposing area targets is to account for variabilities such as undershoot and possible overshoot. If a target itself is an area inclusive of all the variants, the notion of undershoot or overshoot would not make sense, as a phonetic output cannot conceptually be both inside and outside a target at the same time.

There are already some empirical data suggesting that a phonetic target behaves like an asymptote, which can be approached but not exceeded. Nelson et al. (1984) asked subjects to either silently wag their jaws repeatedly, or say “sa sa sa…”, in both cases going from a very slow rate to as fast as possible. Toward the fast end (i.e., over 120 ms/cycle), both the wagging and the sa-sa-sa movements show a positive correlation between cycle duration and movement size. Toward the slow end, however, both tasks show a clear asymptote, i.e., leveling off at a particular displacement level as movement time is longer than 120 ms. What is remarkable is that the sa-sa-sa asymptote is much lower than the wagging asymptote, indicating that the /a/ target has a jaw opening specification much narrower than the maximal range of jaw opening. Because no formant measurements were reported by the study, however, it is not known whether the asymptote effect is also reflected in the speech signal.

### Economy of Effort: The Stress–Stiffness Enigma

**FIGURE 1:**
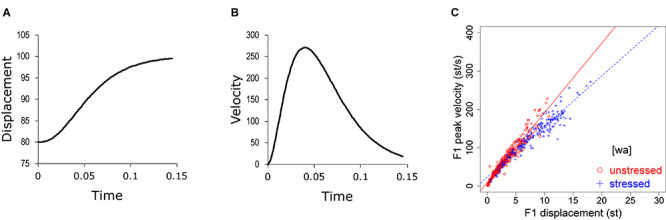
**(A,B)** Displacement **(A)** and velocity **(B)** of a simple movement from 80 toward a target of 100, generated by the second-order version of Eq. (3), to be explained in the section “Interpretation Based on Modeling.” **(C)** Peak velocity over displacement for F1, taken from Figure 6 in the present paper.

The issue of target overshoot is also related to the problem of how economy of effort can be measured. It is not easy to estimate the total muscular activities involved in speech articulation, and no effort to our knowledge has been made to do so. It is possible, however, to estimate articulatory effort by analyzing the kinematics of articulatory movement. Assuming that an articulatory gesture is a movement toward a phonetic target (Lindblom, 1963; Saltzman and Munhall, 1989; Xu and Wang, 2001), articulatory displacement as a function of time should exhibit a trajectory similar to the one shown in Figure 1A, which consists of an initial acceleration phase and a final deceleration phase (Nelson, 1983). The time-varying velocity profile of such a movement should show a unimodal shape (Nelson, 1983; Sorensen and Gafos, 2016), as shown in Figure 1B. Nelson (1983) suggests that the peak of such a velocity profile is a good indicator of effort. Peak velocity has been measured in a number of studies (Kelso et al., 1985; Ostry and Munhall, 1985; Vatikiotis-Bateson and Kelso, 1993; Hertrich and Ackermann, 1997; Xu and Sun, 2002; Xu and Wang, 2009). But a common finding is that its closest correlate is movement amplitude (profile height in Figure 1A). In fact, the two are almost linearly related: the larger the displacement, the greater the peak velocity. An example is given in Figure 1C, which is taken from the present data shown in Figure 6. This quasi-linear relation is true whether the measurement is articulatory, i.e., lips in Hertrich and Ackermann (1997), jaw and lips in Kelso et al. (1985), tongue dorsum in Ostry and Munhall (1985), lips and jaw in Vatikiotis-Bateson and Kelso (1993), or acoustic F_0_, as in Xu and Sun (2002) and Xu and Wang (2009). This means that peak velocity cannot directly tell us about articulatory effort, because it is heavily confounded by the amplitude of the corresponding movement. However, the linear relationship also means that the slope of the regression line between peak velocity and movement amplitude may serve as an indicator of effort: the steeper the slope, the greater the underlying articulatory force. Indeed, this slope, measured as the *v*_p_/*d* ratio (peak velocity over displacement), has been referred to as an indicator of gestural stiffness (Kelso et al., 1985; Ostry and Munhall, 1985).

When using the *v*_p_/*d* ratio as an indicator of effort, however, a puzzle has emerged. That is, the ratio is repeatedly found to be greater in unstressed syllables than in stressed syllables (Ostry et al., 1983; Kelso et al., 1985; Ostry and Munhall, 1985; Beckman and Edwards, 1992; Vatikiotis-Bateson and Kelso, 1993), as can also be seen in Figure 1C. This finding is hard to reconcile with the notion that stressed and unstressed segments vary along a hyper–hypo-articulation dimension (Lindblom, 1990; de Jong et al., 1993; de Jong, 1995; Wouters and Macon, 2002; Janse et al., 2003). This dilemma has been noticed by some studies (Edwards et al., 1991; de Jong et al., 1993; de Jong, 1995), but no consistent solution has been proposed.

In a dynamical system, the fullness of target attainment is jointly affected by stiffness and movement duration. That is, given a stiffness level, the longer the movement duration, the more closely the target is approached by the end of the movement; likewise, given a movement duration, the greater the stiffness, the better the target is attained by the end of the movement. Relating this back to the issue of hyperarticulation: would it be possible that a stressed syllable is often long enough to potentially lead to target overshoot but that there is a restraint on the increase of articulatory force that prevents it? This idea, however, does not seem to be compatible with the fundamental premise of economy of effort and has not been seriously contemplated so far.

### Maximum Rate of Information – An Alternative Principle

The literature review so far suggests that at least some of the assumptions behind the principle of economy of effort are open to question. It is doubtful that speakers always stay safely away from dynamic extremes such as maximum speed of articulatory movement. It is also doubtful that target overshoot is an articulatory means of achieving stress and clarity. Most critically, a solution is overdue for the enigma that stress is associated with *lower* rather than *higher* measured stiffness. As an alternative, here we would like to propose that, in speech production, there is a higher priority than the need to conserve energy: a pressure to transmit as much information as possible in a given amount of time. This can be referred to as the principle of *maximum rate of information*. This principle contrasts with *economy of effort* in a number of ways:

1. *Economy of effort* assumes that speech articulation is an effortful activity akin to running or climbing stairs, and so energy saving is of high priority. *Maximum rate of information* assumes, instead, that energy consumption is negligible in speech production, as speakers are not easily tired out even by talking for hours on end.
2. *Maximum rate of information* assumes that a hard limit in speech production is maximum speed of articulatory movement, which is determined by the total number of muscle fibers that can be recruited for any particular gesture. As a result, undershoot occurs when a gesture is given either too little time or too little effort. It is only in the latter case that undershoot can be reduced by increased effort. In contrast, *economy of effort* assumes that undershoot can always be reduced by applying extra effort.
3. *Maximum rate of information* assumes that phonetic targets are fully specified, invariant goals, and that target approximation is the only mechanism for achieving these goals. As a result, as time elapses during target approximation, articulatory movements naturally slow down as the target is approached. If there is excessive time, as in the case of stress, articulation may be slowed down further to avoid overshoot. In contrast, *economy of effort*, at least its H&H version, implies that overshoot is a possible strategy in the case of stress to enhance contrasts between phonetic categories.
4. *Maximum rate of information* recognizes that duration is a critical encoding dimension, and lengthening and shortening are used as major cues for marking certain communicative functions. The interaction of these durational “targets” with segmental and tonal targets results in both undershoot and gestural slowdown, which is the root of the stress–stiffness enigma. With no assumption about durational targets, *economy of effort* provides no solution to the stress–stiffness enigma.

There is little doubt that, as a communication system, human speech is highly efficient (Hockett, 1960). A strong case is made in the seminal work of Liberman et al. (1967), which recounts the many efforts in the early 60s to develop a coding system to convert printed text to non-speech sounds that could be used by blind people. It turned out that none of the systems developed exceeded the transmission rate of Morse code. Yet the transmission rate of Morse code was only slightly higher than 10% of human speech. Liberman et al. (1967) attributed the efficiency of speech to human’s remarkable ability to perceptually decode coarticulation, and this interpretation eventually led to the motor theory of speech perception (Liberman and Mattingly, 1985). But it ought to be recognized that the efficiency of coding has to be first rooted in speech production, as coarticulation is first and foremost an articulation phenomenon. In addition, coarticulation can be only one of the reasons why speech coding is so efficient, as at least the speed of articulation also needs to be fast enough. The maximization of the rate of information transmission, therefore, may be the ultimate driving force behind many phenomena in speech, including, in particular, both undershoot and effort reduction.

### The Present Study

As can be seen from the foregoing discussion, the popular notion of *economy of effort* predicts that dynamic limits of articulation are seldom approached, because “in normal speech the production system is rarely driven to its limits” (Lindblom, 1983: 219), in order to save energy. It further predicts that extra articulatory effort is made only in the case of stress, for the sake of enhancing phonetic contrasts. Exactly the opposite predictions are made, however, by the principle of *maximum rate of information*. That is, dynamic limits of articulation are frequently approached during normal speech, because articulation is often made as fast as possible, especially in the case of unstressed syllables. In the case of stressed syllables, articulation would actually slow down as the phonetic target is approached in order to prevent overshoot in the face of the extra duration assigned to the stressed syllables.

The goal of the present study is to explore which is more likely the fundamental driving force behind the articulatory dynamics of speech: *economy of effort* or *maximum rate of information*. We will try to answer three specific questions based on the competing predictions mentioned above through an examination of formant movement dynamics: (a) Is the maximum speed of segmental articulation and henceforth the maximum articulatory effort used in meaningful utterances? (b) Are stressed or unstressed syllables more likely to involve the maximum speed of articulation and the associated maximum articulatory effort? And (c) what is the likely articulatory mechanism underlying these dynamic patterns?

Our general approach consists of three parts. The first is a method taken from Xu and Sun (2002), i.e., to ask speakers to imitate resynthesized speech that has been accelerated to a rate that is unlikely to be humanly attainable. The maximum speed of articulation that the participating speakers manage to achieve is therefore treated as an estimate of their voluntary dynamic limits. In the second part, these estimated dynamic limits are compared to the speed of articulation measured in meaningful sentences produced by the same speakers to establish whether and when the maximum speed of articulation is approached in real speech. These two parts will therefore answer the first two research questions. In the third part, we will address the third research question through analysis-by-modeling based on a variable-order dynamical system. The model used will be based on previous work on computational modeling of laryngeal and supralaryngeal articulation (Ostry et al., 1983; Saltzman and Munhall, 1989; Prom-on et al., 2009; Birkholz et al., 2011).

Unlike in most other studies on articulatory dynamics (Kelso et al., 1985; Ostry and Munhall, 1985; Vatikiotis-Bateson and Kelso, 1993; Hertrich and Ackermann, 1997), the kinematic measurements obtained in the present study are those of formants, as was done in many early studies of speech dynamics, including, in particular, Lindblom (1963) and Moon and Lindblom (1994), that have led to the H&H theory. Because of the popular assumption that articulatory movements should ideally be studied by examining articulatory data only, the following justifications are given to explain why formant measurements can also provide highly relevant information about articulatory dynamics.

Given that listeners hear speech through acoustics, all the perceptually relevant articulatory movements are reflected in the acoustic output. Among the acoustic properties, formant movements have been shown to be perceptually relevant since classic works like Cooper et al. (1952) and Liberman et al. (1954). Formant synthesis systems like the Klatt synthesizers (Klatt, 1980, 1987), though low on naturalness compared to the state-of-the-art speech technology today, have achieved high intelligibility (Taylor, 2009). The widely accepted source-filter theory of speech production (Fant, 1960; Stevens, 1998) has established that the acoustic properties of speech sounds, especially those of the vowels, are determined by the shape of the entire vocal tract, which consists of not only the articulators that are easily measured (e.g., tongue tip, tongue blade, tongue dorsum, and lips), but those that are less accessible, like the tongue root, the pharynx, and even the larynx (Hoole and Kroos, 1998; Demolin et al., 2000). Thus, the movement of any particular articulator is not for its own sake but only as part of a whole movement that achieves a set of overall aerodynamic and acoustic effects. Those acoustic effects are arguably the ultimate goal of a phonetic target (Mattingly, 1990; Johnson et al., 1993b; Hanson and Stevens, 2002; Perrier and Fuchs, 2015; Whalen et al., 2018). In contrast, specific articulatory kinematic measurements can provide only a partial approximation of the goal-oriented articulatory movements as a whole (Whalen et al., 2018).

In fact, Hertrich and Ackermann (1997) and Perkell et al. (2002), after careful examination of articulatory dynamics, both suggested that the phonetically most relevant information may be found in the acoustic signal. Noiray et al. (2014) find that acoustic patterns faithfully reflect even highly idiosyncratic articulatory patterns that carry crucial information for perceptual contrast. Whalen et al. (2018) further demonstrate that cross-speaker variability in acoustics and articulation is closely related, rather than articulation being more variable than acoustics, as previously argued (Johnson et al., 1993a). Furthermore, the perturbation theory (Fant, 1980; Stevens, 1998) would predict that only the lowest formants (mostly F1–F3) are directly controllable by deliberate maneuvers of movable articulators such as the tongue and jaw, because too many nodes and antinodes are associated with higher formants to make it possible to deform the vocal tract shape at all the right locations without canceling out each other’s perturbation effects. This means that most of the contrastive vowel information can only be carried by the first few formants. Therefore, formant trajectories are arguably a better indicator of articulatory dynamics, because they reflect vocal tract shapes as a whole, including those parts that are hard to measure, and they are fewer in number. This would make formant measurements such as its displacement, velocity, the *v*_p_/*d* ratio, etc., no less valid than those of any particular articulator.

Formant data are not without limitations, however. A well-known issue is the sometimes abrupt shift of affiliation of the second and third formants with resonance cavity as the vocal tract shape changes smoothly, e.g., between [i] and [a] (Bailly, 1993; Stevens, 1998). When this happens, the continuity of formant movements may be affected. Furthermore, formant trajectories do not capture the spectral patterns between the formants, which may also be phonetically relevant (Ito et al., 2001). For the purpose of the present study, however, the relevance of formant trajectories can be tested by examining whether their kinematics show similar patterns as those of articulatory movements. At least for fundamental frequency, highly linear relations between F_0_ velocity and F_0_ movement amplitude have been found (Xu and Sun, 2002; Xu and Wang, 2009), which resemble the linear relations in articulatory or limb movement (Kelso et al., 1985; Ostry and Munhall, 1985; Vatikiotis-Bateson and Kelso, 1993; Hertrich and Ackermann, 1997). This is despite the fact that F_0_ is the output of a highly complex laryngeal system (Zemlin, 1988; Honda, 1995). Whether formant kinematics also exhibit similar linear relations and thus warrant the kinematic analyses that have been applied to limb and F_0_ movements will therefore be an empirical question. More importantly, as a fundamental principle of any empirical investigation, the most critical requirement is to always make minimal contrast comparisons (Gelfer et al., 1989; Boyce et al., 1991) so that any potential adverse effects are applicable to both the experimental and reference conditions. This will also be the principle that guides the design of the present study.

## Materials and Methods

### Stimuli

The stimuli were resynthesized target syllable sequences to be imitated or printed sentences to be read aloud, as presented below. To guarantee continuous formant tracking, we used CV syllables where the consonants are glides and the vowels have maximally different vocal tract shapes from the adjacent glides. A further advantage of using glides instead of obstruent consonants is that they present the least amount of gestural overlap between C and V because glides, as semivowels, are specified for the entire shape of the vocal tract rather than mainly at the place of articulation as in obstruent consonants. The lack of gestural overlap should maximize time pressure. A similar strategy was adopted by Moon and Lindblom (1994) for the same reason. To assess whether the maximum speed of articulation is approached during speech, we asked the same group of subjects to produce meaningful sentences in which the same glide–vowel syllables are embedded.

#### Glide–Vowel Sequences

**FIGURE 2:**
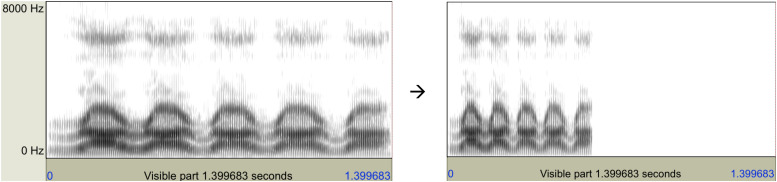
**Left:** Spectrogram of /rarararara/ spoken by author XY at a normal speech rate. **Right:** Spectrogram of the same utterance speeded up with Praat by a factor of 2.

There were five CV sequences, each consisting of five identical glide–vowel syllables, as shown below. They were first spoken by author YX at a normal rate in a sound-treated booth. They were then resynthesized using the Pitch Synchronous Overlap and Add (PSOLA) algorithm implemented in Praat (Boersma, 2001) to increase the mean syllable rate to 8 syllables per second, which exceeds the fastest repetitive rate for glide–vowel syllables reported previously (Siguard, 1973; Tiffany, 1980). As an example, Figure 2 shows the spectrograms of the original and accelerated rarararara sequence.

1. wawawawawa
2. yayayayayaya
3. wiwiwiwiwi
4. yoyoyoyoyo
5. rarararara

#### Sentences

The stimulus sentences, as shown below, contain symmetrical CVC patterns that each resemble a single cycle of a repetitive CVC sequence. These CVC patterns all appear in the first word of a two-word noun phrase. This is to guarantee that they are not subject to phrase-final lengthening (Nakatani et al., 1981). Each pattern is placed in a stressed syllable and an unstressed syllable in two different sentences. The unstressed /waw/ appears in three positions, early, middle, and late, for examining possible positional differences (not performed in the present study). All other patterns appear only in the sentence-medial position. The boldfaced syllables in the target words are stressed. These stress placements are natural to the native speakers, and subjects had no difficulty producing the intended stress patterns.

1. So Ba**ba**li Street will overlay Wa**we**cka Place.
2. They say Wa**we**cka Place is close to Ba**ba**li Street.
3. I have to say Wa**we**cka Place is better.
4. I went to the **Wa**wa Company for the results.
5. My brother saw “**Ya**-Ya Sisterhood” on Friday.
6. I just saw Ya**yo**na Parker at the movie.
7. People say **Wee**-Wee brand is the best.
8. This is the day Wea**wea**la Company opens.
9. I saw a **yo**-yo string in the park.
10. I got a yo**yo**logy book at the library.
11. You can see the au**ro**ra lights in the North.
12. People say Ra**re**tta King is getting famous.

### Subjects and Recording Procedure

Fifteen speakers of American English, 8 females and 7 males, age 18–25 years, participated as subjects. They were undergraduate students at Northwestern University or other universities in the Chicago area. All subjects signed informed consent approved by the Northwestern University Institutional Review Board and were paid for their participation.

The subject sat in front of a computer screen wearing a head-mounted condenser microphone (Countryman Isomax hypercardiod). During the recording, the stimuli were displayed on a web page controlled by a Javascript program. The program randomized the stimulus order so that each subject read a different random list. Another program, SoundEdit, ran in the background on the same computer to digitize the acoustic signal directly onto the hard disk at a 22.05 kHz sampling rate and 16-bit resolution.

For the syllable sequences, in the slow condition, the subject read aloud each sequence at the rate of careful speech; in the other two conditions, during each trial, the subject listened to a model sequence and then immediately imitated the sequence in two ways: (1) as fast as possible five times without slurring, and (2) as exaggeratedly as possible another three times without slurring. For the sentences, the subject was instructed to say each sentence first at a normal rate three times and then at the fastest rate possible another three times without slurring. The experimenter, who was a native speaker of American English, made sure that the target words were all said with the right stress patterns.

### Measurements

**FIGURE 3:**
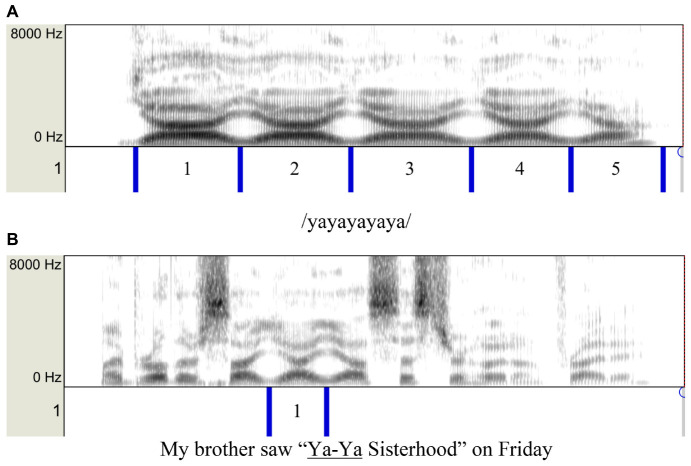
**(A)** A sample spectrogram of /yayayayaya/ with manually placed segmentation. **(B)** A sample spectrogram of a sentence containing /yay/ with manually placed segmentation.

The first step in taking the measurements was to demarcate the syllables, as illustrated in Figure 3. The demarcation points were set at the extrema of either F1 or F2 formant tracks. The procedure was facilitated by a Praat script (a predecessor of FormantPro: Xu and Gao, 2018) that cycled through all the utterances produced by each speaker and displayed the waveform, spectrogram, and a TextGrid for inserting the demarcation points and labeling the syllables. The demarcation points were first set manually and then corrected by the script based on the LPC formant tracks, which were smoothed by a trimming algorithm that eliminated abrupt bumps and sharp edges (originally developed for trimming F_0_ contours: Xu, 1999).

For the /wawawawawa/, /yayayayaya/, and /rarararara/ sequences, the demarcation points were set at the F1 minima, as illustrated in Figure 3A. For the other two sequences, because of the small F1 movements, the demarcation points were set at the F2 minima (for /wiwiwiwiwi/) or maxima (for /yoyoyoyoyo/). For the sentences, only the target syllables were demarcated, as shown in Figure 3B.

Based on the demarcation of the syllables, the following measurements were taken.

max*F*_j_ (*st*) – highest value in the *j*th formant in semitones in each unidirectional formant movement, where *j* = 1, 2, 3. The conversion from Hz to semitones was done with the equation:

(1) st=12logf2j

where *f*_j_ is the formant value in Hz. Note that, here, the reference value for *f*_j_ is assumed to be 1 Hz.

min*F*_j_ (*st*) – lowest value in the *j*th formant in each unidirectional formant movement.

*F*_j_-displacement (onset and offset) – formant difference (in *st*) between adjacent max*F*_j_ and min*F*_j_. There are two unidirectional movements in each syllable: one for the onset ramp of the formant movement toward the vowel target, and the other for the offset ramp. Thus for each syllable, two displacements were computed.

mean *F*_j_-displacement – average of onset and offset displacements.

movement duration (onset and offset) – time interval between adjacent formant maximum and minimum.

syllable duration – sum of onset and offset movement durations in each syllable.

peak velocity (onset and offset) – positive and negative extrema in the velocity curve corresponding to the rising and falling ramps of each unidirectional formant movement. The velocity curves were computed by taking the first derivative of formant curves. Following Hertrich and Ackermann (1997), the formant curves were low-pass filtered at 20 Hz with the Smooth command in Praat, but the velocity curves themselves were not smoothed so as not to reduce the magnitude of peak velocity.

*v*_p_/*d* ratio (onset and offset) – ratio of peak velocity to displacement calculated as the slope of the linear regression of peak velocity over displacement across all the points in a unidirectional formant movement.

### Analysis

The first goal of the analysis is to determine whether the production of meaningful utterances has approached various dynamic limits observed in nonsense syllable sequences. This is assessed in two ways. The first is to compare the sequence conditions and the sentence conditions in terms of the distribution of formant displacement as a function of movement duration. The comparison is made with the theoretical bounds defined by Nelson (1983) as a reference to see if the distributions show patterns that suggest that speakers may indeed have maximized their articulatory effort. The second is to make the comparisons in terms of peak velocity as a function of displacement: *v*_p_/*d*. If much similarity is found between the sequence conditions and the sentence conditions for the same articulatory movement, this would again be an indication that a dynamic limit of articulation is approached in sentence production.

The second goal of the analysis is to determine whether the dynamic limits are more likely approached during stressed or unstressed syllables in the sentence condition. This will be done with both formant displacement as a function of movement duration and peak velocity as a function of movement amplitude.

#### Displacement Over Duration

**FIGURE 4:**
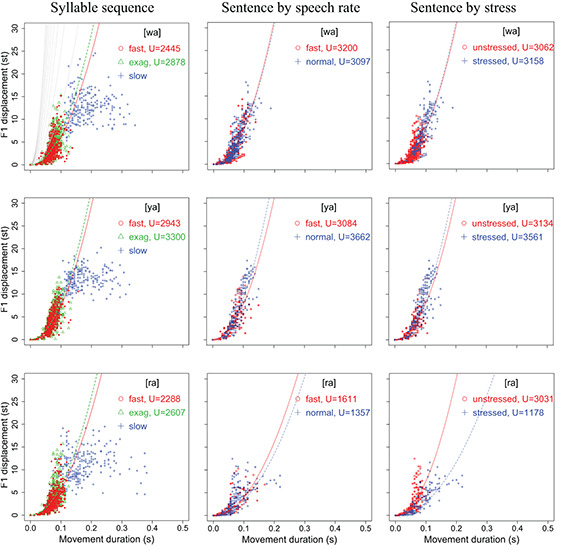
Scatter plots of F1 displacement over movement duration for [wa], [ya], and [ra] in the syllable sequences (column 1) and sentences (columns 2,3) produced by all 15 speakers (with 3 speakers missing in the slow sequence condition). In each plot, the parabolic curves represent theoretical minimum-time limits for frictionless movements with constant acceleration–deceleration magnitudes based on Nelson (1983). See text for detailed explanation.

**FIGURE 5:**
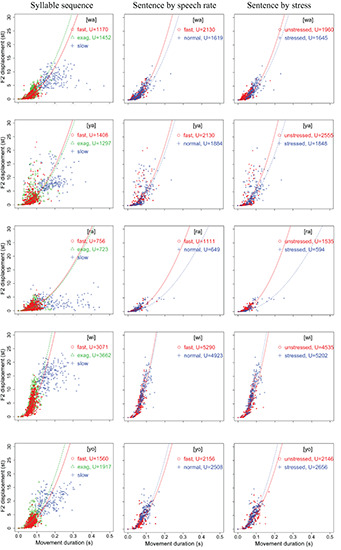
Scatter plots of F2 displacement over movement duration for [wa], [ya], [ra], [wi], and [yo] in the syllable sequences (column 1) and sentences (columns 2,3) produced by all 15 speakers (with 3 speakers missing in the slow sequence condition). In each plot, the parabolic curves represent theoretical minimum-time limits for frictionless movements with constant acceleration–deceleration magnitudes based on Nelson (1983). See text for detailed explanation.

Figures 4, 5 display scatter plots of F1 and F2 displacement over movement duration for [wa], [ya], [ra], [wi], and [yo] in the syllable sequences (column 1) and sentences (columns 2, 3) produced by all 15 speakers (except for the slow sequence condition, for which three speakers were not recorded). In Figure 4, F1 is not plotted for [wi] and [yo] because the formant movements were often too small to allow reliable location of their maxima or minima. In column 1 of both figures, the points are separated into the three speaking modes for the syllable sequences: fast, exaggerated without slowing down, and slow. In column 2, the points are separated by speech rate in the sentence condition, and in column 3, they are separated by word stress in the sentence condition.

In terms of the vertical distribution of the (T, D) points, column 1 of both Figures 4, 5 shows a three-way split across the three conditions, with the fast rate closest to the bottom and the slow rate closest to the top, although there is much overlap between the three conditions. In column 2 of both figures, the distributions are very similar to those of the fast and exaggerated conditions in column 1, indicating that the same syllables in the sentences are spoken with a similar amount of muscle force. The plots in column 2 also show that there is no clear vertical separation between normal and fast speaking rates, which contrasts with column 3, where a better separation can be seen between stressed and unstressed syllables. Unsurprisingly, stressed syllables have larger formant displacements than unstressed syllables.

In the top-left graph of Figure 4, we have plotted the gray parabolic curves generated by Eq. (2), where *T*_*m*_ is a function of *U*, which is a *theoretical* physical force (acceleration) determined by the maximum amount of muscle force that can be exerted for a movement (Nelson, 1983). The curves therefore represent theoretical minimum-time bounds given specific values of *U*. According to Nelson (1983: 140), given a particular time bound, all physically realizable movements have to lie to the right of that bound, and “any movement having a distance-time (D, T) point on or to the left of a particular contour would require a peak acceleration greater than the value for that contour.” While the bounds can be theoretically moved left by increasing the value of *U*, the cost of such an increase would rise rapidly, as indicated by the closer spacing of the contours as they shift leftward. Thus, there is bound to be a physical limit that is virtually impossible to cross.

(2) $$T_{m}=2\left(D/U\right){}^{1/2}$$

In Nelson (1983), the unit of *U* is physical distance in meters. Here, in the top-left plot of Figure 4, the theoretical bounds correspond to *U* = 5,000, 10,000, 15,000, …, 50,000, and are arbitrarily set to be above the bounds for most of the formant values in Figures 4, 5. To assess the amount of muscle force exerted during the articulation of the target utterances, we fitted Eq. (2) to the (T, D) points in each condition for an optimal value of *U* with the fitModel function in the R package TIMP (Mullen and van Stokkum, 2007). The fitted curves are shown in each plot in Figures 4, 5. With these fitted curves, the (T, D) distribution in different conditions can be compared for their *U* values.

For the syllable sequences, the fitting is done only for the fast and exaggerated conditions, because the slow condition shows a ceiling effect as syllable duration becomes increasingly long. As can be seen in the top-left graph of Figure 4, the F1 points in the slow condition do not parallel any of the time bounds but are largely horizontally distributed. This indicates that formant displacement ceases to consistently increase as movement duration goes beyond around 0.125 s (125 ms). This asymptotic distribution resembles those in Figure 5 of Nelson et al. (1984: 950), with similarity even in terms of the critical duration of around 120 ms.

With the fitted curves, we can compare the values of *U* in different conditions for an initial assessment of the relative articulatory force applied by the speakers. For the syllable sequences in column 1, *U* is always greater in the exaggerated than in the fast syllable sequences, except for F2 in [ya] and [ra]. This seems to be consistent with the instructions given to the subjects in terms of speech mode. For the most crucial question of the current study, namely, whether syllables are spoken in sentences as fast as in sequences, as shown in both Figures 4, 5, in the majority of the cases, the values of *U* in sentences are actually greater than those in sequences, with the exception of [ra]. In the case of [ra], for some reason, both F1 and F2 have relatively smaller ranges of displacement in sentences than in sequences. In terms of relative articulatory force in the sentence condition, however, there is no consistent pattern based on either speech rate or stress, although there is a tendency toward greater force for fast rate than for normal rate.

Overall, analysis of the distribution of displacement over duration (D, T) shows that CVC syllables spoken in sentences were articulated with at least as much muscle force as meaningless syllable sequences. However, the relative articulatory force in different sentence conditions is not yet clear. For that, we will turn to the analysis of peak velocity, which has been associated more directly with articulatory force (Nelson et al., 1984; Kelso et al., 1985; Ostry and Munhall, 1985; Perkell et al., 2002).

#### Peak Velocity Over Displacement (*v*_p_/*d* Ratio)

**FIGURE 6:**
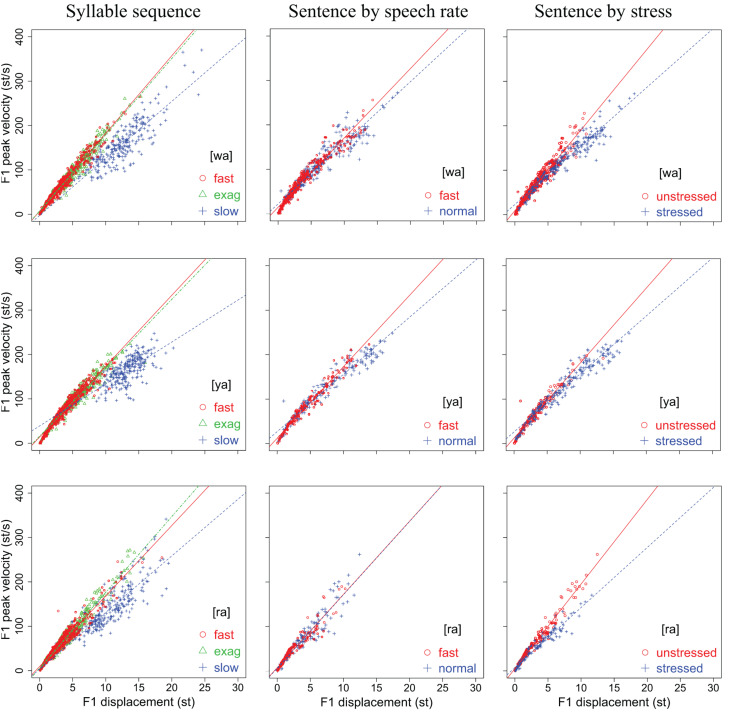
Scatter plots of F1 peak velocity over displacement for [wa], [ya], and [ra] in the syllable sequences (column 1) and sentences (columns 2,3) produced by all 15 speakers. Linear regression lines are fitted to each rate or stress condition. See text for detailed explanation.

**FIGURE 7:**
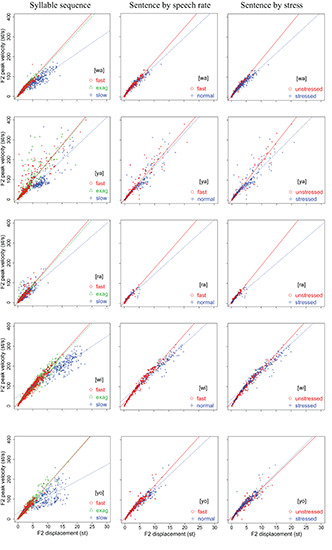
Scatter plots of F2 peak velocity over displacement for [wa], [ya], [ra], [wi], and [yo] in the syllable sequences (column 1) and sentences (columns 2,3) produced by all 15 speakers. Linear regression lines are fitted to each rate or stress condition. See text for detailed explanation.

Figures 6, 7 display scatter plots of peak formant velocity over formant displacement for [wa], [ya], [ra], [wi], and [yo] in the syllable sequences (column 1) and sentences (columns 2, 3) produced by all 15 speakers (except for the slow condition in the syllable sequences, for which there are no data for three of the speakers). Because most distributions are highly linear (as found for articulatory movements: Ostry et al., 1983; Kelso et al., 1985; Ostry and Munhall, 1985; Beckman and Edwards, 1992; Vatikiotis-Bateson and Kelso, 1993), linear regression lines are fitted for every group of data to obtain the *v*_p_/*d* ratio.

In column 1, the slope of the regression line is much shallower in the slow sequences than in the fast and exaggerated sequences, but the differences between the fast and exaggerated sequences are rather small. A two-way repeated measures ANOVA with *v*_p_/*d* ratio as the dependent variable and rate and syllable as independent variables showed significant effects of rate on F1 [*F*(2,22) = 105.99, *p* < 0.0001] and F2 [*F*(2,22) = 90.36, *p* < 0.0001] and significant effects of syllable on F1 [*F*(4,44) = 2.8136, *p* = 0.0365] and F2 [*F*(4,44) = 2.852, *p* = 0.0347] (with three speakers missing in the slow sequence condition). A Bonferroni/Dunn *post hoc* test showed significant differences between slow and both fast and exaggerated conditions but not between the latter two. This is true of both F1 and F2.

**FIGURE 8:**
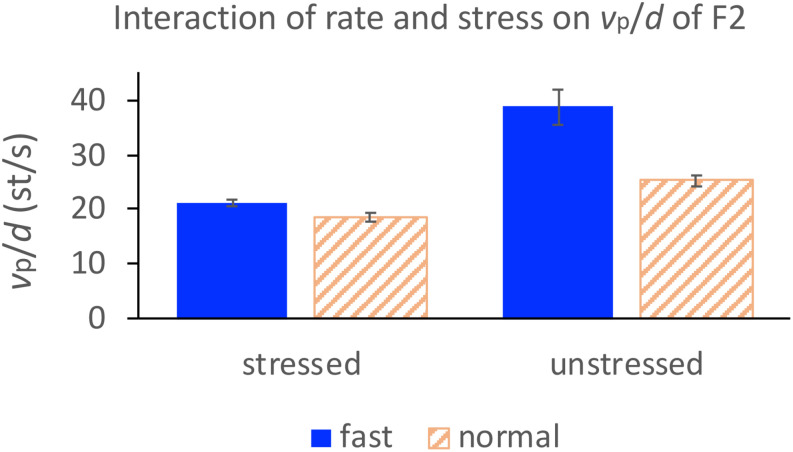
Interaction of rate and stress on *v*_p_/*d* for F2 in the sentence condition.

In column 2, the regression slopes are consistently steeper for fast rate than for normal rate, which is not surprising. What is striking is that in column 3, the regression slopes are consistently steeper for the unstressed syllables than for the stressed syllables. A two-way repeated measures ANOVA with *v*_p_/*d* ratio as the dependent variable and rate and stress as independent variables showed significant effects of rate on F1 [*F*(1,14) = 13.66, *p* = 0.0024] and F2 [*F*(1,14) = 17.42, *p* = 0.0009] and significant effects of stress on F1 [*F*(1,14) = 4.86, *p* = 0.0448] and F2 [*F*(1,14) = 70.97, *p* < 0.0001]. For F2, there is also a significant interaction between rate and stress due to the much larger difference between stressed and unstressed syllables at fast rate than at slow rate, as shown in Figure 8. From Figure 8, it is clear that the greatest *v*_p_/*d* values are from unstressed syllables at fast rate. As can be seen in Table 1, this is the condition where syllable duration (79.9 ms) has dropped well below the critical duration of 120 ms mentioned in the section “Displacement Over Duration.”

**TABLE 1:** Syllable duration in ms in the sentence condition, with standard deviations in parenthesis.

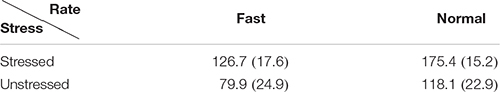

Overall, the difference between stressed and unstressed syllables, as shown in column 2, is quite similar to that between the two fast rates shown in column 1. This can be further seen in Tables 2, 3, which show the mean *v*_p_/*d* ratios in syllable sequences and sentences, respectively. These differences were compared by performing two-tailed paired *t*-tests between the syllable sequences and sentences; the results are shown in Table 4. Either unstressed syllables had significantly greater *v*_p_/*d* ratios than the sequences (F2 in all conditions), or there were no significant differences (F1 in fast sentences). For stressed syllables, there was no difference in either formant when sentences were at the fast rate. At normal rate, stressed syllables had significantly different *v*_p_/*d* ratios from the sequences but always with *lower* values. Overall, then, *v*_p_/*d* is no lower in sentences than in sequences unless the syllable is stressed and at the normal speech rate.

**TABLE 2:** Mean *v*_p_/*d* ratio in syllable sequences, with standard deviations in parentheses.

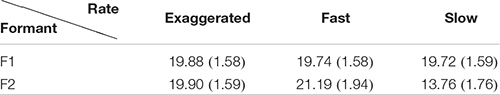

**TABLE 3:** Mean *v*_p_/*d* ratio in sentences, with standard deviations in parentheses.

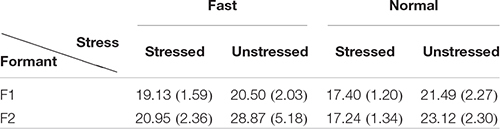

**TABLE 4:** Two-tailed paired *t*-test comparisons between mean *v*_p_/*d* ratios in syllable sequences and in sentences.

			**F1**		**F2**	
**Sequence**	**Sentence**		***p***	**Difference**	***p***	**Difference**
Exaggerated	Fast	Stressed	0.0788		0.1066	
		Unstressed	0.2246		<0.0001	–8.97
	Normal	Stressed	0.0001	2.48	<0.0001	2.66
		Unstressed	0.0128	–1.61	0.0001	–3.21
Fast	Fast	Stressed	0.1230		0.6268	
		Unstressed	0.1043		<0.0001	–7.68
	Normal	Stressed	0.0002	2.34	<0.0001	3.95
		Unstressed	0.0050	–1.7465	0.0096	–1.9252

*The differences (sequence−speech) between the two conditions where *p* < 0.05 are shown.*

These results therefore show that syllables in meaningful sentences are spoken with *v*_p_/*d* ratios that are equal to or even greater than those in nonsense sequences, except when they are stressed and at normal rate. Assuming that *v*_p_/*d* is a reliable indicator of gestural stiffness, CVC syllables spoken in sentences are articulated with at least as much muscle force as the fastest meaningless syllable sequences. On the other hand, within the sentence condition, the finding of greater *v*_p_/*d* ratios in unstressed syllables than in stressed syllables has only deepened the mystery of the stress–stiffness enigma. Looking at the plots in Figures 6, 7 again, it is mostly those points with large displacements that are “bent down” relative to the linear regression lines, and these seem to have reduced the regression slopes. This is true in both the sequence and sentence conditions. In the next section, we will use computational modeling to explore whether this is a potential source of the stress–stiffness enigma.

### Interpretation Based on Modeling

Various models have been proposed based on either acoustic or articulatory data to account for the articulatory dynamics underlying articulatory effort. In Lindblom (1963) and Moon and Lindblom (1994), a numerical model was used to simulate undershoot by representing formant values at turning points using a decaying exponential function. The model is based on the kinematics of the movement (single displacement or velocity measurement per movement) rather than its dynamics (continuous displacement and velocity trajectories). Such a strategy, however, is suboptimal in modeling (Kelso et al., 1985), because it is developed for simulating only particular kinematic measurements and so are unable to simulate the continuous trajectories of articulatory or acoustic movements. Moon and Lindblom (1994) also proposed a dynamic model. However, it is not a target-approaching model because each movement is simulated as consisting of an onset phase in the direction of the muscle force and an offset phase in the opposite direction [also see Fujisaki et al. (2005) for a similar strategy]. Such movements are thus more complex than the unidirectional movement with a unimodal velocity profile described above (Nelson, 1983). Also, in the model, the effect of stiffness is the opposite of the more widely accepted conceptualization, namely, higher stiffness should lead to greater displacement. We will therefore not consider those two types of models.

A more common approach is to use a dynamical system such as a linear mass-spring model to simulate simple movements with a unimodal velocity profile like the one illustrated in Figures 1A,B, in which displacement as a function of time exhibits a unidirectional asymptotic trajectory toward the equilibrium point of the system (Nelson, 1983; Ostry et al., 1983; Kelso et al., 1985). The equilibrium point serves as an attractor toward which the system converges over time regardless of its initial state (Kelso et al., 1986; Saltzman and Munhall, 1989). Such progressive convergence is clearly seen in the F_0_ contours of a tone when preceded by different tones (Xu, 1997, 1999). This tonal convergence behavior has led to the Target Approximation model (Xu and Wang, 2001) and its quantitative implementation, quantitative target approximation (qTA), which is a critically damped third-order system driven by pitch targets as forcing functions (Prom-on et al., 2009). These dynamic models, however, have not yet been used to simulate kinematic patterns as was done in Lindblom (1963) and Moon and Lindblom (1994) (except in a limited way in Ostry and Munhall, 1985). In the present study, we will explore the ability of dynamic models to simulate observed kinematic measurements and, in the process, explore answers to questions about dynamic constraints in speech, as follows:

1. What gives rise to the observed quasi-linear *v*_p_/*d* function?
2. Is stiffness near maximum in speech, or is it kept well off the ceiling?
3. Why is the slope of the *v*_p_/*d* function steeper in the unstressed syllables than in the stressed syllables?

#### A Generalized Target Approximation Model

The model we are using is a generalized target approximation model extended from the qTA model (Prom-on et al., 2009). Like many other systems (Ostry et al., 1983; Kelso et al., 1985; Saltzman and Munhall, 1989), it is a mass-spring system that generates movement trajectories by sequentially approaching successive phonetic goals in an asymptotic manner. But unlike the others, it is a system with variable order to allow the simulation of different levels of complexity of the interactions among the variables. Mathematically, the target approximation movement can be represented by a general *N-*th order model:

(3) $$y\,\left(t\right)=x\,\left(t\right)+e^{-\text{λ}\,t}\,\sum_{k=0}^{N-1}c_{k}\,t^{k}$$

where *x*(*t*) is the linear target function,

(4) x⁢(t)=m⁢t+b

The target in this context is different from those in other mass-spring models where the equilibrium is a fixed displacement value (Feldman, 1986; Saltzman and Munhall, 1989; Perrier et al., 1996b). *m* and *b* represent the slope and height of the target function, respectively. This linear function is motivated by findings of dynamic tones in tone languages (Xu and Wang, 2001) and diphthongs in English (Gay, 1968). When the target is static, i.e., *m* = 0, as is assumed in all the calculations in the present study, the linear function in Eq. (4) is equivalent to an equilibrium point as in other mass-spring models. λ is related to stiffness (equivalent of ${\text{ω}}_{n}=\sqrt{\frac{k}{m}}$, where *k* is stiffness and *m* is mass in a mass-spring-dashpot system). The coefficients *c*_*k*_ are determined from initial conditions and target parameters:

(5) $$c_{k}=\left\{ \begin{array}{c} y\,\left(0\right)-b,k=0\\ y^{k}\,\left(0\right)+c_{0}\,\lambda -m,k=1\\ \frac{1}{k!}\,\left(y^{k}\,\left(0\right)-\sum_{i=0}^{k-1}\frac{k!}{\left(k-i\right)!}\,c_{i}\,{\left(-\lambda \right)}^{k-i}\right),k\geq 2 \end{array}\right.$$

In this general model, as in its third-order predecessor, articulatory state is assumed to be transferred across movement boundaries, i.e., from the end of the current movement to the beginning of the next movement. For example, in the case of qTA, three articulatory states are transferred across movement boundaries: displacement, velocity, and acceleration. As the order of the model increases, higher-order articulatory states are also transferred.

**FIGURE 9:**
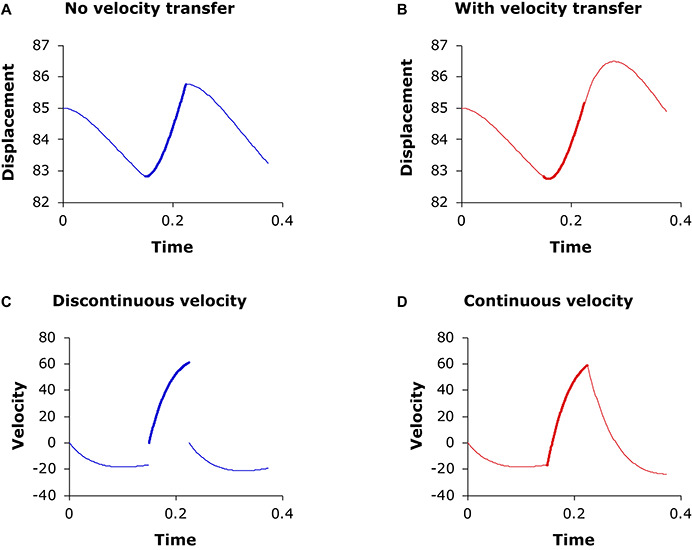
**(A)** Three consecutive target approximation movements (*d*_1_ = 0.15, *d*_1_ = 0.075, *d*_1_ = 0.15) with continuous displacement at junctions (where line thickness changes) but without continuous velocity, so that velocity always restarts from 0 at the beginning of each movement, as shown in panel **(C)**. **(B)** Three consecutive target approximation movements where velocity is continuous at the junctions, as shown in **(D)**. Generated by the second-order version of Eq. (3) with *λ* = 20, *b*_1_ = 80, *b*_2_ = 100, *b*_3_ = 80.

The cross-boundary state transfer is important not only because it guarantees the smoothness of the trajectory at the boundary but also because it fully simulates the higher-order carryover influences of one movement on the next, which has been found to sometimes even exceed that due to cross-boundary displacement transfer (Chen and Xu, 2006). This is illustrated in Figure 9 with the second-order version of Eq. (3). In Figure 9A, the three adjacent movements have continuous displacement at their junctions (where the line thickness changes) but not continuous velocity (as shown in Figure 9C). The displacement function in Figure 9B is smoother than that in Figure 9A because its first derivative is also continuous at the junctions, as shown in Figure 9D. The movement amplitude in Figure 9B is larger than in Figure 9A because the high velocity at the end of the second movement has delayed the turning point into the third movement (the second movement has smaller amplitude in Figure 9B than in Figure 9A because it first has to overcome the negative velocity transferred from the end of the first movement when trying to achieve its higher target). As is apparent from Figure 9, whether higher-order state transfer is implemented makes a significant difference in terms of measured (as opposed to intended) movement duration, displacement, and peak velocity as well as other, derived measurements.

#### Simulation and Interpretation

A program was written in C to generate a sequence of three movements based on the generalized model (Eq. 3). In all of the simulations, the following parameter settings were kept constant:

*m*_1_ = *m*_2_ = *m*_3_ = 0 (target slope)
*y*_01_ = 85 (initial displacement)
*b*_1_ = 80, *b*_2_ = 100, *b*_3_ = 80 (target height)
*λ*_1_ = *λ*_2_ = *λ*_3_ (rate of target approximation)
*d*_1_ = 0.2, *d*_2_ = 0.1, *d*_3_ = 0.3 (duration of target approximation).

The units of these parameters are arbitrary, but the values were chosen so that the output would be numerically comparable to the data shown in Figures 6, 7.

Three parameters were systematically varied in the simulation: *k*, λ, and *d*_2_, where λ = λ_1_ = λ_2_ = λ_3_.

**FIGURE 10:**
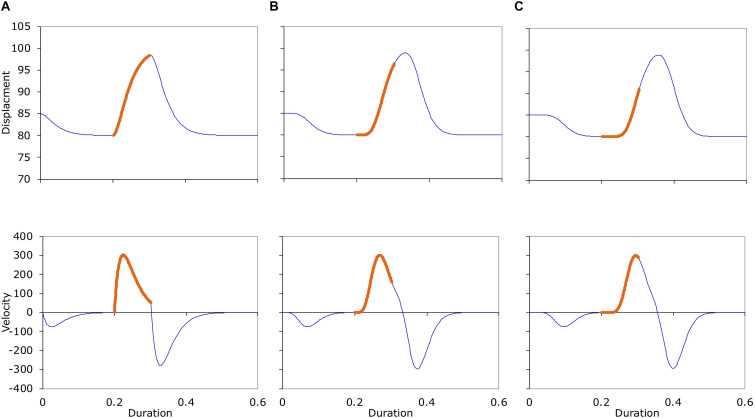
Top: Displacement trajectories of three consecutive target approximation movement sequences generated with the **(A)** 2nd, **(B)** 8th, and **(C)** 14th order versions of Eq. (3). Shifts in line thickness are where target change occurs. Bottom: Velocity profiles of the three movement sequences in the top row. See text for parameters used to generate the trajectories.

Figure 10 shows the displacement (top) and velocity (bottom) trajectories of three sequences, with model orders of 2nd (A), 8th (B), and 14th (C). The thick section in the middle of each trajectory corresponds to the approximation interval of the second target, whose ideal displacement is *b*_2_ = 100 and duration is *d*_2_ = 0.1. As can be seen, as the model order increases, the amount of delay in the target approximation in the displacement trajectory also increases, the velocity profiles become more and more symmetrical, and the velocity peak occurs increasingly later in the target interval.

The target intervals as shown in Figure 10 are invisible in real speech data, of course. Actual measurements can therefore be based only on visible landmarks such as turning points. We therefore followed common practice and took the following measurements from the displacement and velocity trajectories, regardless of the actual target intervals used in generating the trajectories.

Displacement – difference in height between the first and second turning point in the displacement trajectory.

Movement duration – horizontal distance between the first and second turning points.

Peak velocity – peak value in the velocity trajectory.

**FIGURE 11:**
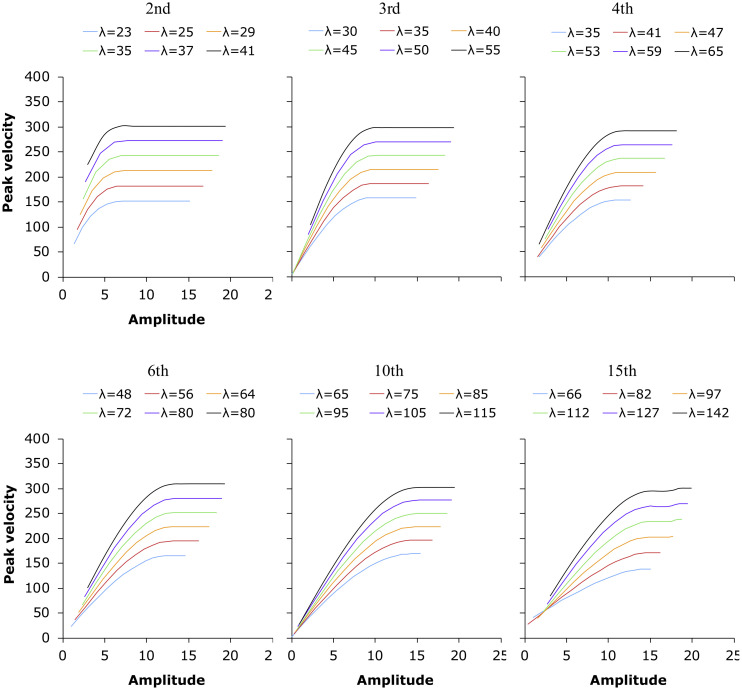
Peak velocity as a function of movement amplitude for movement trajectories generated by Eq. (3) at six different orders and various stiffness levels represented by λ.

With these measurements, we plotted peak velocity as a function of displacement, as shown in Figure 11.

With the plots in Figure 11, we can now attempt to answer the questions raised at the beginning of the modeling section. The first question is what may have given rise to the quasi-linearity of the *v*_p_/*d* function. The first thing to notice is that, regardless of the level of stiffness represented by λ, as displacement increases, peak velocity sooner or later reaches a plateau after the initial rising slope. This contrasts with Figures 6, 7, where no obvious plateaus can be seen at the end of the slopes except a slowdown in the rise of peak velocity in some of the slow conditions in the syllable sequences (column 1 in both figures). However, a closer observation may reveal some resemblances. Looking across the plots in Figure 11, we can see that as the order of the model increases, the rising slopes become longer and shallower, and if we ignore the plateaus for a moment, the initial slopes become increasingly similar to the quasi-linear distributions of *v*_p_/*d* in Figures 6, 7. Furthermore, for any given order, the greater the stiffness, the sooner a plateau is reached as displacement increases.

These two trends can be more clearly seen in Table 5, which lists the minimum durations at which a selection of the peak velocity trajectories in Figure 11 nearly reach a plateau (arbitrarily defined as when the increase in velocity is <1 with each unit of increase in displacement). For each order of the model, three stiffness (λ) levels are shown. From Table 5, we can see that if the underlying mechanism of speech production is assumed to be a target approximation process of some kind, the following conclusions can be made:

**TABLE 5:** Critical duration of quasi-linear *v*_p_/*d* at different stiffness levels: durations at which a peak velocity trajectory increases <1 with each unit of increase in displacement.

	**2nd order**			**6th order**			**10th order**		
Stiffness (λ)	23	29	37	48	64	80	65	85	105
Critical duration	80.5	61.7	48.9	130.0	107.0	87.4	143.9	123.1	103.6
Corresponding displacement	8.0	8.6	9.3	13.4	14.6	14.3	14.9	16.1	16.1
Corresponding peak velocity	151.5	212.3	271.9	165.5	223.6	280.4	169.0	223.0	276.3

1. The articulatory system is likely to be a higher rather than a lower order one.
2. Regardless of the order of the system, the stiffness with which the system is operating is such that peak velocity only approaches the maximal level without reaching it.

In other words, the quasi-linear *v*_p_/*d* function shown in Figures 6, 7 could be generated by a critically damped high-order linear system operating at a stiffness level that allows only approximation but not attainment of the underlying target within the allocated duration. This stiffness level should not be interpreted as low, however. Rather, it suggests that the applied muscle force is already at the maximum of the articulatory system but is too weak relative to the meager amount of time allocated to each movement. This is consistent with previous findings that the maximum rate of articulation is often applied even in normal speech (Tiffany, 1980; Xu and Sun, 2002; Kuo et al., 2007; Adank and Janse, 2009). In fact, the target approximation-based *v*_p_/*d* functions shown in Figure 11 suggest that only when articulation is operating with near-maximum stiffness can the measured *v*_p_/*d* show quasi linearity as in Figures 6, 7.

**FIGURE 12:**
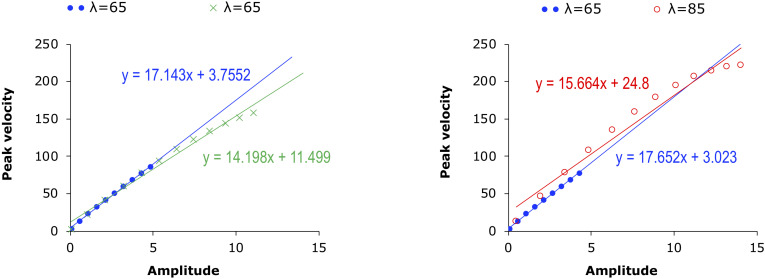
Simulated linear fitting of the data in the 10th order condition in Figure 11. **Left:** Filled circles and the crosses are both points from the contours generated with λ = 65, but the displacement of the circled points is capped at 5 while that of the crossed points is capped at 11. **Right:** Filled circles are points from the contours generated with λ = 65, with the amplitude capped at 5. Open circles are points from the contours generated with λ = 85, with amplitude capped at 14.

If speech is indeed generally produced at its speed limit, the time pressure should be worse for unstressed syllables than for stressed syllables. As shown in Table 1, unstressed syllables, even spoken at normal rate, are shorter than stressed syllables spoken at fast rate. With such short duration, most of the target approximation movements are cut short or truncated. This means that the *v*_p_/*d* points measured in unstressed syllables tend to be mostly located in the lower-left portions of the *v*_p_/*d* function shown in Figure 11. The effect of this is illustrated in Figure 12, which replots some of the 10th order curves from Figure 11. In the left graph, the two curves are both for the condition where λ = 65, but they differ in their data range. The range of the circled points is *d* ≤ 5, while that of the crossed points is *d* ≤ 11. When both of them are linearly fitted, the slope of the linear function for the points with the smaller range is steeper than that for those with the larger range: 17.143 vs. 14.198. Thus the greater steepness of the slope of the linearly fitted *v*_p_/*d* function for unstressed syllables could be due to truncation of the associated movements under time pressure. This truncation effect can sometimes even make a movement with greater stiffness appear to have low stiffness, as illustrated in the right graph of Figure 12. There, the range of the function with λ = 65 is again *d* ≤ 5, but the range of the points with higher stiffness (λ = 85) is *d* ≤ 14. The linear fitting of the two functions now shows a steeper slope for the points with lower stiffness than for the points with greater stiffness. Thus, measurement of *v*_p_/*d* as a linear function is heavily dependent on the range of displacement values being fitted: the smaller the range, the greater the likely value of *v*_p_/*d*, other things being equal.

**FIGURE 13:**
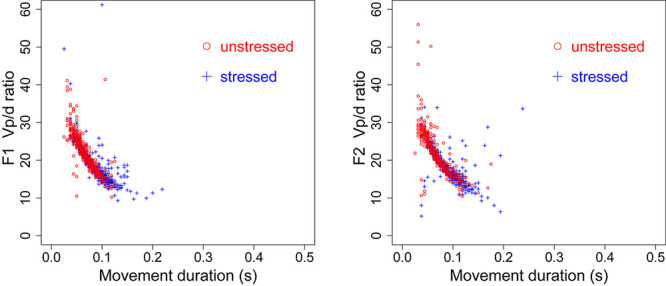
*v*_p_/*d* as a function of movement duration for both F1 **(left)** and F2 **(right)** by all speakers.

As for whether the left or right graph is the likely scenario in the case of the stress–stiffness enigma, Table 6 shows maximum displacements of F1 and F2 in stressed and unstressed syllables at both speech rates from the present data. Although stressed syllables show consistently greater displacements than unstressed syllables, the differences are not extremely large. This means that the underlying stiffness may not be drastically different. This indeed seems to be the case, as shown in Figure 13, where the movement-specific *v*_p_/*d* ratio in the current data is plotted as a function of the duration for both F1 and F2. The stressed and unstressed syllables seem to share the same function of movement-specific *v*_p_/*d* relative to duration regardless of their differential distributions in duration. This suggests that the left graph of Figure 12 is the more likely scenario.

**TABLE 6:** Maximum displacement in *st* in the sentence condition, with standard deviations in parentheses.

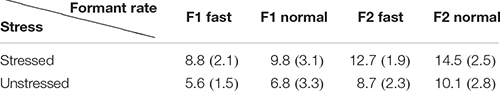

To conclude the modeling section, assuming that articulatory gestures are target approximation movements that can be modeled by a mass-spring system, speakers generally produce gestures too quickly for target approximation to complete even with maximum muscle force, and the time shortage is much *worse* for unstressed syllables than for stressed syllables. It is the incompleteness of the target approximation movements that may have led to the quasi-linearity of the generally observed *v*_p_/*d* function, but the slope of the linearly fitted *v*_p_/*d* function is also inversely related to the range of observable displacements, which tends to be smaller in unstressed syllable than in stressed syllables. This is the likely source of the stress–stiffness enigma.

## Discussion and Conclusion

The experimental and modeling data presented above have provided evidence in support of the principle of *maximum rate of information* as an alternative to the principle of *economy of effort*, based on a test of the competing predictions from the two principles through an examination of formant dynamics. First, in the section “Analysis,” the distribution of formant displacement as a function of movement duration shows that articulatory movements in meaningful speech utterances are no slower than the equivalent movements in meaningless syllable sequences that are produced at fast rate or spoken as exaggeratedly as possible without slowing down. Second, this fast speed in articulatory movement is confirmed by *v*_p_/*d*, peak velocity as a function of displacement, a measurement that has been considered as an indicator of gestural stiffness. This stiffness, however, is shown to be consistently higher for unstressed syllables than for stressed syllables, similar to the findings of previous studies based on articulatory data. Third, the modeling simulation in the section “Interpretation Based on Modeling” provides evidence that (a) the widely found linearity of the peak velocity over displacement function is likely due to stiffness being too low relative to the temporal intervals allocated to individual target approximation movements, and (b) the shortage of time is more severe for unstressed than for stressed syllables, and this may have led to *v*_p_/*d* being consistently greater for unstressed syllables than for stressed syllables. Overall, therefore, speech seems to be generally operating at a near-ceiling level as far as stiffness is concerned. As a result, there is probably little or no room for speakers to further increase stiffness when undershoot happens.

These results, therefore, are incompatible with the principle of economy of effort, especially in the form of the H&H theory (Lindblom, 1990), which assumes that there is always room for further strengthening of articulatory effort to achieve hyper-articulation. On the contrary, the present results, together with many similar findings discussed earlier, are more consistent with Lindblom’s (1963) earlier undershoot model, which recognizes shortage of time as a major source of incomplete target attainment. From the perspective of *maximum rate of information*, the highest priority in speech production is to transmit as much information as possible in a given amount of time. The most precious resource for speech would therefore be time rather than energy. Unstressed syllables are given less time because they are less important than stressed syllables and can therefore afford to have greater undershoot.

**FIGURE 14:**
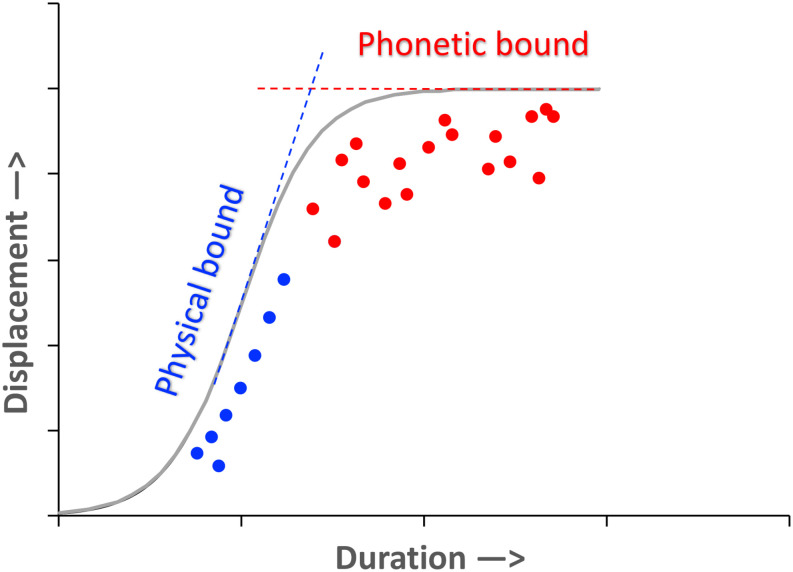
Hypothetical physical and phonetic bounds to displacement as a function of movement time (duration). The curved portion connecting the two bounds is arbitrarily drawn with no existing supporting data. The data points are also arbitrary, and are for illustration purposes only.

Shortage/abundance of time is not the only factor that determines measured stiffness in articulatory movements. Another factor is the need for articulatory precision. In motor movement research, it is well known that a more accurate movement takes a longer time to execute (Fitts, 1954; Schmidt et al., 1979; Soechting, 1984). In speech, phonetic categories require high precision to assure their perceptual recognition. The precision requirement is so high that children do not achieve an adult level of performance until their teens (Lee et al., 1999). This high precision must be associated with highly precise targets, and maintaining this target also means not to overshoot them even when there is enough time. This idea is illustrated in Figure 14. There, the vertical bound represents the physical limit in terms of how much time is needed to perform a movement of any particular amplitude (which may differ widely across speakers: Tiffany, 1980). For movements that are given abundant time, however, there is also a phonetic bound specified by the acoustic properties of the sound, as represented by the high plateau in Figure 14. This phonetic bound acts like a ceiling that prevents speakers from overshooting the target. From the perspective of an information system, fidelity of transmission is an essential property of its capacity (Shannon, 1948), and assuring precision of target attainment for stressed syllables is therefore consistent with the principle of maximum rate of information. Note, however, that sometimes a phonetic bound can lie beyond a physical bound. In the case of an alveolar stop, for example, the target of the tongue tip can be set beyond the surface of the alveolar ridge. This would guarantee an air-tight seal during closure (Löfqvist and Gracco, 1999).

The modeling analysis in the section “Interpretation Based on Modeling” has suggested a solution to the enigma that stress is associated with *lower* rather than *higher* measured stiffness (Ostry et al., 1983; Kelso et al., 1985; Ostry and Munhall, 1985; Perkell et al., 2002). As illustrated in Figure 12, the widely reported steeper slope of the *v*_p_/*d* function for unstressed syllables than for stressed syllables is likely due to a measurement bias arising from the short duration of unstressed syllables in general. This short duration results in a truncation of the target approximation movement so that, typically, only the fast-rising portion of the *v*_p_/*d* function is included in the data, which would have resulted in a linearly fitted *v*_p_/*d* indicating a greater stiffness than the underlying stiffness. On the other hand, for stressed syllables, because they are more likely to be given a longer time for target approximation, more of the final tapering off of the *v*_p_/*d* function is likely included. This would have resulted in a linearly fitted *v*_p_/*d* indicating a lower stiffness than the underlying stiffness.

Also, in light of the analysis and modeling in the present study, it becomes clear that none of the measurements we have examined here, namely, displacement, peak velocity, *v*_p_/*d* ratio, and movement-specific *v*_p_/*d* ratio, can be treated as a direct indicator of articulatory effort. Articulatory effort can be meaningfully assessed only when all the known factors are effectively controlled, and some kind of quantitative model of articulation is applied. A further caveat is that the simulation of formant dynamics done in the present study is not meant to be a simulation of full articulatory dynamics. Nor can the simulation of the dynamics of any single articulator achieve that goal. More realistic simulation can be done only through full-scale articulatory synthesis, as tested in some of our recent studies (Prom-on et al., 2013, 2014; Xu et al., 2019).

In conclusion, the findings of the present study have provided support for the principle of maximum rate of information in speech production. Under this principle, speech is generally produced at an overall maximum rate of articulation, due to which many of the syllables and segments are subject to undershoot because of lack of time, and the undershoot is much more severe in unstressed syllables than in stressed syllables. The high rate of undershoot in unstressed syllables may have led to a tendency for their measured stiffness in terms of *v*_p_/*d* ratio to be unduly high, as suggested by our modeling analysis. In cases where more time is given, as in the case of stressed syllables, the precision of target approximation, as required for the fidelity of information transmission, results in a reduced rate of increase in peak velocity as a function of displacement, as demonstrated by our modeling analysis. This may have led to a tendency for their measured stiffness in terms of *v*_p_/*d* ratio to be unduly low.

## Data Availability Statement

The datasets generated for this study are available on request to the corresponding author.

## Author Contributions

YX conceived and designed the study, conducted the experiments, performed the computation modeling, and wrote the manuscript. SP-O wrote the modeling program, and reviewed and edited the manuscript.

## Conflict of Interest

The authors declare that the research was conducted in the absence of any commercial or financial relationships that could be construed as a potential conflict of interest.
