# Corrigendum: Economy of Effort or Maximum Rate of Information? Exploring Basic Principles of Articulatory Dynamics

**DOI:** 10.3389/fpsyg.2020.01753

**Published:** 2020-07-28

**Authors:** Yi Xu, Santitham Prom-on

**Affiliations:** ^1^Department of Speech, Hearing and Phonetic Sciences, University College London, London, United Kingdom; ^2^Department of Computer Engineering, King Mongkut's University of Technology Thonburi, Bangkok, Thailand

**Keywords:** maximum rate of information, economy of effort, stiffness, peak velocity, target approximation

In the original article, there were two errors in Equation (5). The lower limit of the summation should be *i* = 0 rather than *i* = 1. The denominator in the fraction should be (*k* – *i*)! rather than (*N* – *k*)!

A correction has been made to Equation (5). The corrected equation appears below.

(5) $$\begin{array}{l} c_{k}=\{\begin{array}{ll} y\left(0\right)-b, & k=0\\ y^{k}\left(0\right)+c_{0}\lambda -m, & k=1\\ \frac{1}{k!}\;(y^{k}\left(0\right)-\sum_{i=0}^{k-1}\frac{k!}{\left(k-i\right)!}c_{i}{\left(-\lambda \right)}^{k-i}), & k\geq 2 \end{array} \end{array}$$

The authors apologize for this error and state that this does not change the scientific conclusions of the article in any way. The original article has been updated.

